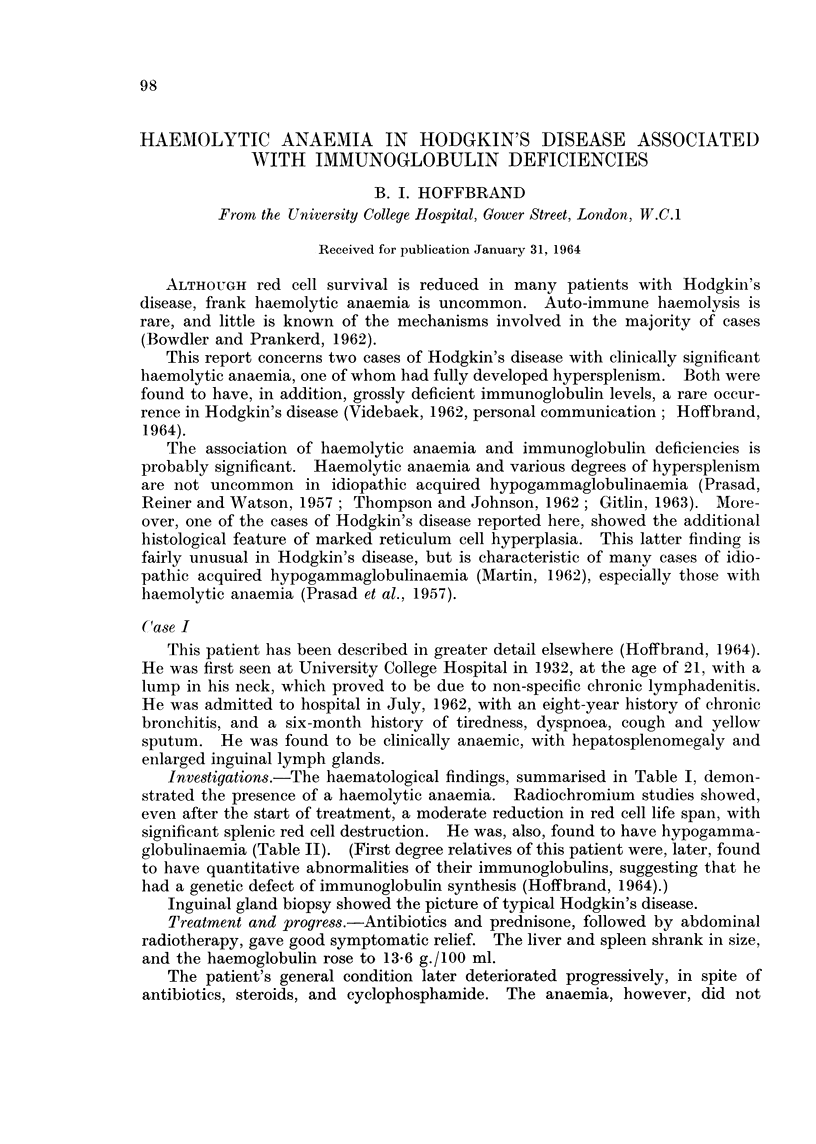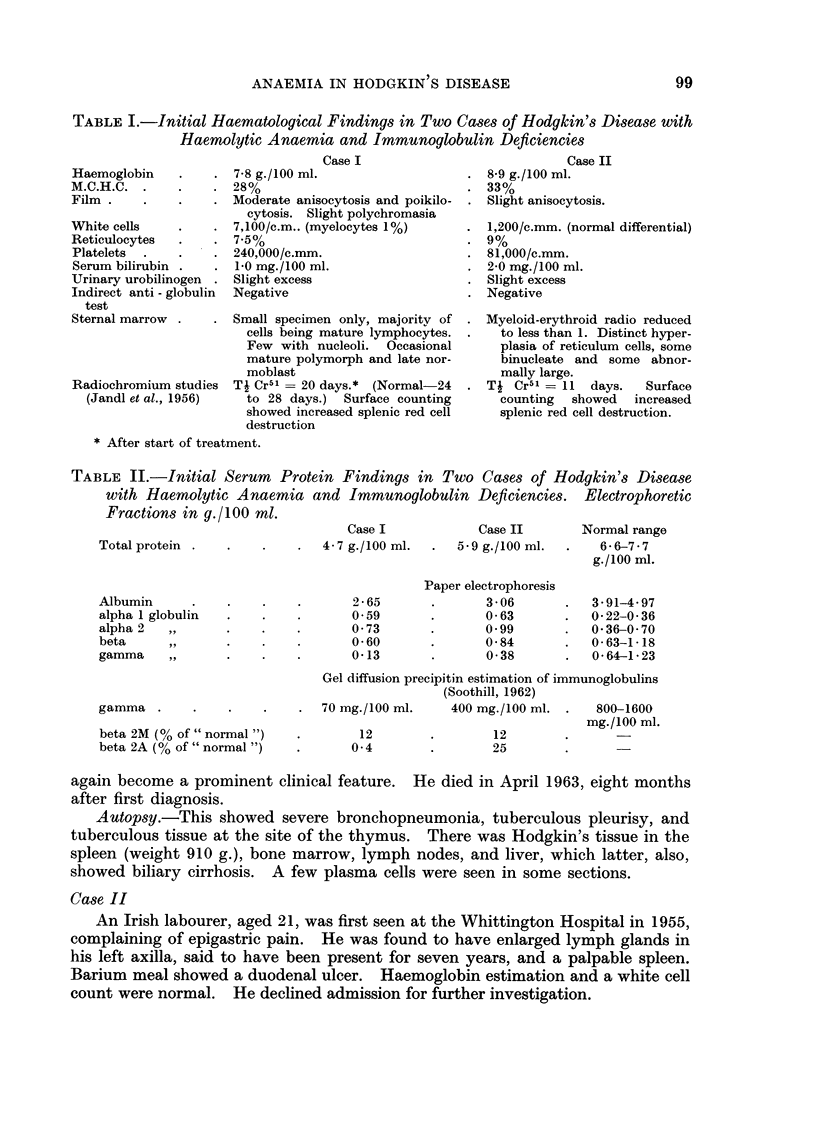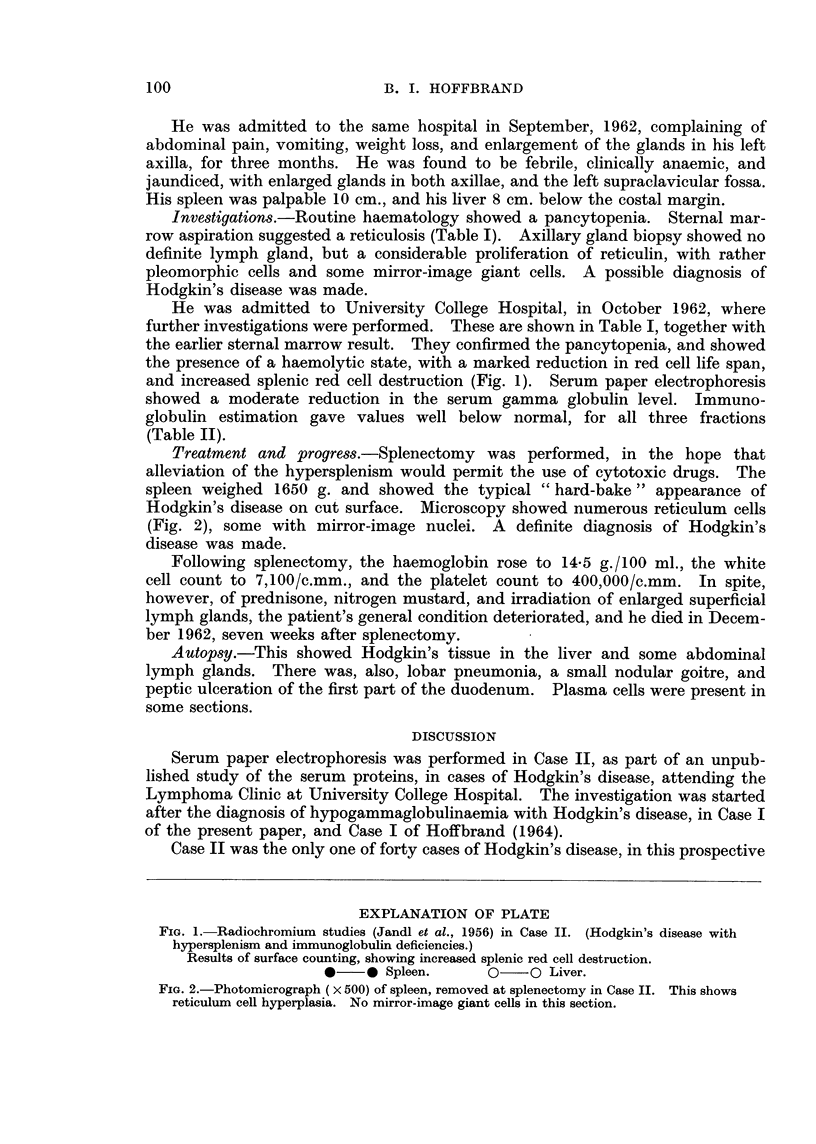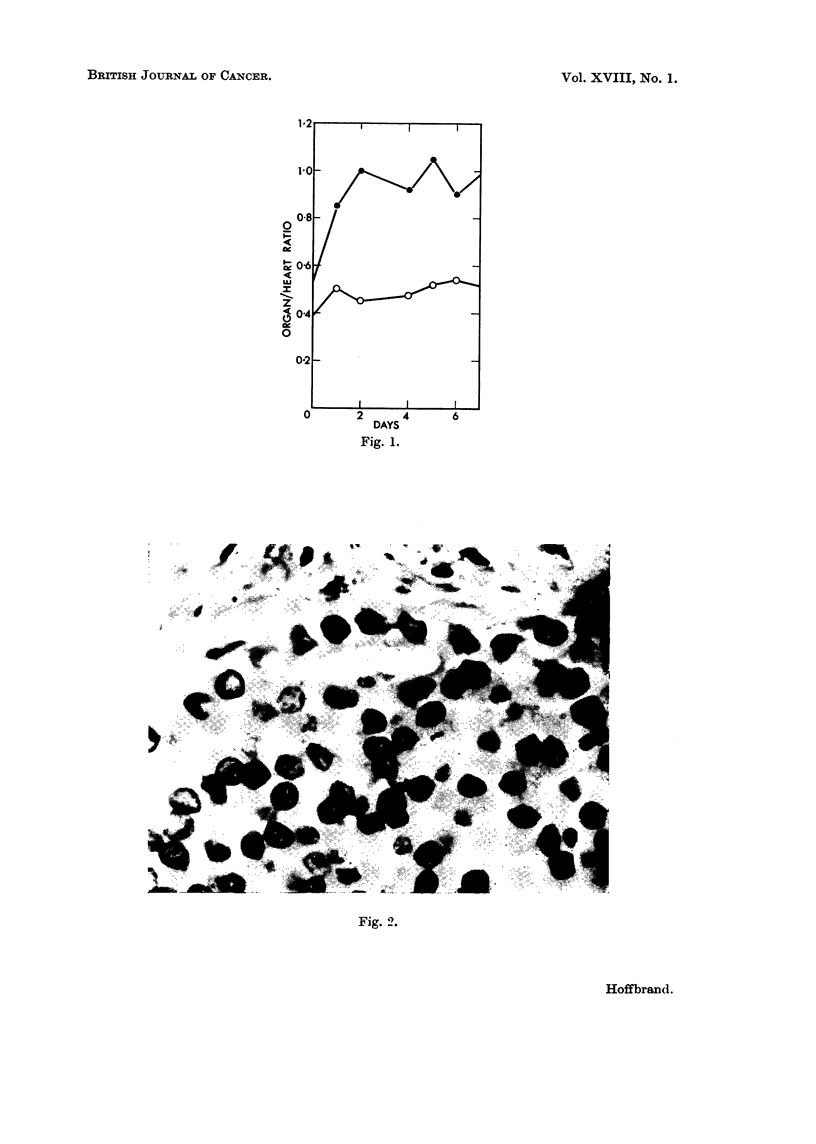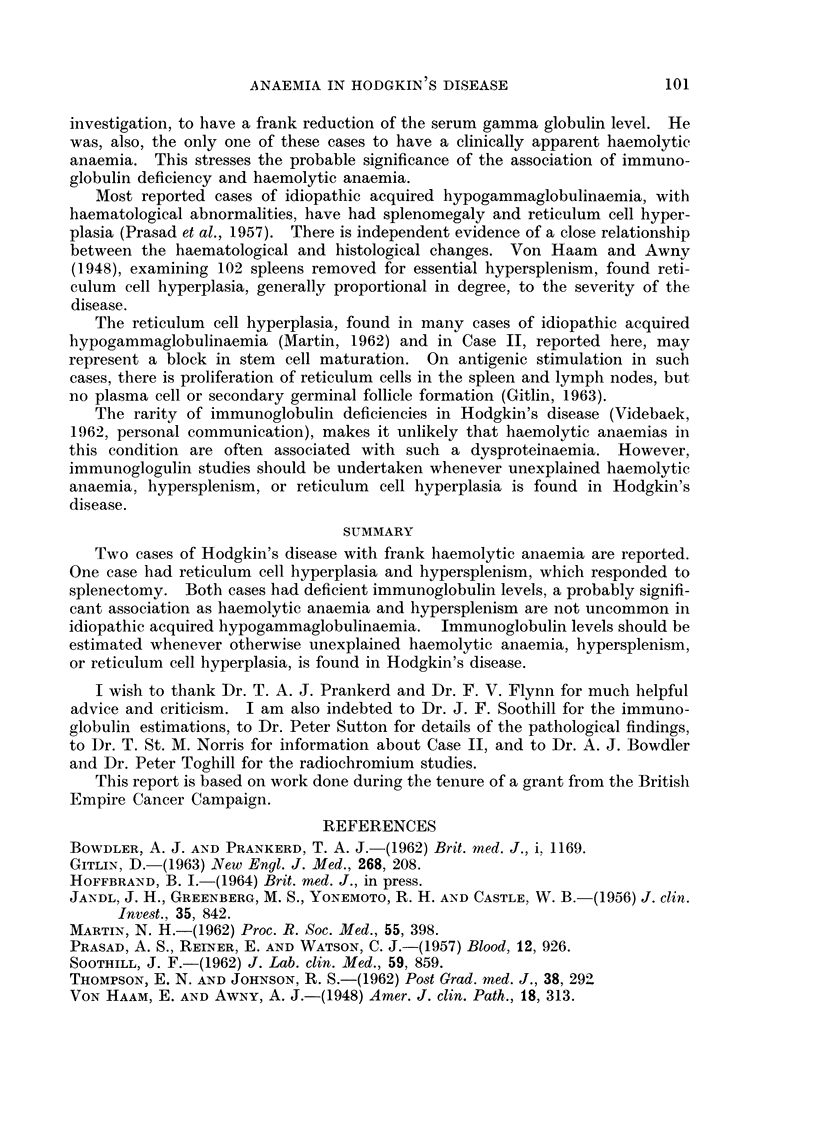# Haemolytic Anaemia in Hodgkin's Disease Associated with Immunoglobulin Deficiencies

**DOI:** 10.1038/bjc.1964.9

**Published:** 1964-03

**Authors:** B. I. Hoffbrand

## Abstract

**Images:**


### HAEMOLYTIC ANAEMIA IN HODGKIN'S DISEASE ASSOCIATED WITH IMMUNOGLOBULIN DEFICIENCIES

B. I. HOFFBRAND

*From the University College Hospital, Gower Street, London, W.C.*1

Received for publication January 31, 1964

ALTHOUGH red cell survival is reduced in many patients with Hodgkin's disease, frank haemolytic anaemia is uncommon. Auto-immune haemolysis is rare, and little is known of the mechanisms involved in the majority of cases (Bowdler and Prankerd, 1962).

This report concerns two cases of Hodgkin's disease with clinically significant haemolytic anaemia, one of whom had fully developed hypersplenism. Both were found to have, in addition, grossly deficient immunoglobulin levels, a rare occurrence in Hodgkin's disease (Videbaek, 1962, personal communication ; Hoffbrand, 1964).

The association of haemolytic anaemia and immunoglobulin deficiencies is probably significant. Haemolytic anaemia and various degrees of hypersplenism are not uncommon in idiopathic acquired hypogammaglobulinaemia (Prasad, Reiner and Watson, 1957 ; Thompson and Johnson, 1962 ; Gitlin, 1963). Moreover, one of the cases of Hodgkin's disease reported here, showed the additional histological feature of marked reticulum cell hyperplasia. This latter finding is fairly unusual in Hodgkin's disease, but is characteristic of many cases of idiopathic acquired hypogammaglobulinaemia (Martin, 1962), especially those with haemolytic anaemia (Prasad *et al.*, 1957).

*Case I*

This patient has been described in greater detail elsewhere (Hoffbrand, 1964). He was first seen at University College Hospital in 1932, at the age of 21, with a lump in his neck, which proved to be due to non-specific chronic lymphadenitis. He was admitted to hospital in July, 1962, with an eight-year history of chronic bronchitis, and a six-month history of tiredness, dyspnoea, cough and yellow sputum. He was found to be clinically anaemic, with hepatosplenomegaly and enlarged inguinal lymph glands.

*Investigations.*—The haematological findings, summarised in Table I, demonstrated the presence of a haemolytic anaemia. Radiochromium studies showed, even after the start of treatment, a moderate reduction in red cell life span, with significant splenic red cell destruction. He was, also, found to have hypogammaglobulinaemia (Table II). (First degree relatives of this patient were, later, found to have quantitative abnormalities of their immunoglobulins, suggesting that he had a genetic defect of immunoglobulin synthesis (Hoffbrand, 1964).)

Inguinal gland biopsy showed the picture of typical Hodgkin's disease.

*Treatment and progress.*—Antibiotics and prednisone, followed by abdominal radiotherapy, gave good symptomatic relief. The liver and spleen shrank in size, and the haemoglobulin rose to 13·6 g./100 ml.

The patient's general condition later deteriorated progressively, in spite of antibiotics, steroids, and cyclophosphamide. The anaemia, however, did not

TABLE I.—*Initial Haematological Findings in Two Cases of Hodgkin's Disease with Haemolytic Anaemia and Immunoglobulin Deficiencies*

| | Case I | Case II |
|---|---|---|
| Haemoglobin | 7·8 g./100 ml. | 8·9 g./100 ml. |
| M.C.H.C. | 28% | 33% |
| Film | Moderate anisocytosis and poikilocytosis. Slight polychromasia | Slight anisocytosis. |
| White cells | 7,100/c.m.. (myelocytes 1%) | 1,200/c.mm. (normal differential) |
| Reticulocytes | 7·5% | 9% |
| Platelets | 240,000/c.mm. | 81,000/c.mm. |
| Serum bilirubin | 1·0 mg./100 ml. | 2·0 mg./100 ml. |
| Urinary urobilinogen | Slight excess | Slight excess |
| Indirect anti-globulin test | Negative | Negative |
| Sternal marrow | Small specimen only, majority of cells being mature lymphocytes. Few with nucleoli. Occasional mature polymorph and late normoblast | Myeloid-erythroid radio reduced to less than 1. Distinct hyperplasia of reticulum cells, some binucleate and some abnormally large. |
| Radiochromium studies (Jandl *et al.*, 1956) | T½ $Cr^{51}$ = 20 days.* (Normal—24 to 28 days.) Surface counting showed increased splenic red cell destruction | T½ $Cr^{51}$ = 11 days. Surface counting showed increased splenic red cell destruction. |

* After start of treatment.

TABLE II.—*Initial Serum Protein Findings in Two Cases of Hodgkin's Disease with Haemolytic Anaemia and Immunoglobulin Deficiencies. Electrophoretic Fractions in g./100 ml.*

| | Case I | Case II | Normal range |
|---|---|---|---|
| Total protein | 4·7 g./100 ml. | 5·9 g./100 ml. | 6·6–7·7 g./100 ml. |
| | | Paper electrophoresis | |
| Albumin | 2·65 | 3·06 | 3·91–4·97 |
| alpha 1 globulin | 0·59 | 0·63 | 0·22–0·36 |
| alpha 2 ,, | 0·73 | 0·99 | 0·36–0·70 |
| beta ,, | 0·60 | 0·84 | 0·63–1·18 |
| gamma ,, | 0·13 | 0·38 | 0·64–1·23 |
| | Gel diffusion precipitin estimation of immunoglobulins (Soothill, 1962) | | |
| gamma | 70 mg./100 ml. | 400 mg./100 ml. | 800–1600 mg./100 ml. |
| beta 2M (% of " normal ") | 12 | 12 | — |
| beta 2A (% of " normal ") | 0·4 | 25 | — |

again become a prominent clinical feature. He died in April 1963, eight months after first diagnosis.

*Autopsy.*—This showed severe bronchopneumonia, tuberculous pleurisy, and tuberculous tissue at the site of the thymus. There was Hodgkin's tissue in the spleen (weight 910 g.), bone marrow, lymph nodes, and liver, which latter, also, showed biliary cirrhosis. A few plasma cells were seen in some sections.

*Case II*

An Irish labourer, aged 21, was first seen at the Whittington Hospital in 1955, complaining of epigastric pain. He was found to have enlarged lymph glands in his left axilla, said to have been present for seven years, and a palpable spleen. Barium meal showed a duodenal ulcer. Haemoglobin estimation and a white cell count were normal. He declined admission for further investigation.

He was admitted to the same hospital in September, 1962, complaining of abdominal pain, vomiting, weight loss, and enlargement of the glands in his left axilla, for three months. He was found to be febrile, clinically anaemic, and jaundiced, with enlarged glands in both axillae, and the left supraclavicular fossa. His spleen was palpable 10 cm., and his liver 8 cm. below the costal margin.

*Investigations.*—Routine haematology showed a pancytopenia. Sternal marrow aspiration suggested a reticulosis (Table I). Axillary gland biopsy showed no definite lymph gland, but a considerable proliferation of reticulin, with rather pleomorphic cells and some mirror-image giant cells. A possible diagnosis of Hodgkin's disease was made.

He was admitted to University College Hospital, in October 1962, where further investigations were performed. These are shown in Table I, together with the earlier sternal marrow result. They confirmed the pancytopenia, and showed the presence of a haemolytic state, with a marked reduction in red cell life span, and increased splenic red cell destruction (Fig. 1). Serum paper electrophoresis showed a moderate reduction in the serum gamma globulin level. Immunoglobulin estimation gave values well below normal, for all three fractions (Table II).

*Treatment and progress.*—Splenectomy was performed, in the hope that alleviation of the hypersplenism would permit the use of cytotoxic drugs. The spleen weighed 1650 g. and showed the typical " hard-bake " appearance of Hodgkin's disease on cut surface. Microscopy showed numerous reticulum cells (Fig. 2), some with mirror-image nuclei. A definite diagnosis of Hodgkin's disease was made.

Following splenectomy, the haemoglobin rose to 14·5 g./100 ml., the white cell count to 7,100/c.mm., and the platelet count to 400,000/c.mm. In spite, however, of prednisone, nitrogen mustard, and irradiation of enlarged superficial lymph glands, the patient's general condition deteriorated, and he died in December 1962, seven weeks after splenectomy.

*Autopsy.*—This showed Hodgkin's tissue in the liver and some abdominal lymph glands. There was, also, lobar pneumonia, a small nodular goitre, and peptic ulceration of the first part of the duodenum. Plasma cells were present in some sections.

### DISCUSSION

Serum paper electrophoresis was performed in Case II, as part of an unpublished study of the serum proteins, in cases of Hodgkin's disease, attending the Lymphoma Clinic at University College Hospital. The investigation was started after the diagnosis of hypogammaglobulinaemia with Hodgkin's disease, in Case I of the present paper, and Case I of Hoffbrand (1964).

Case II was the only one of forty cases of Hodgkin's disease, in this prospective

---

### EXPLANATION OF PLATE

FIG. 1.—Radiochromium studies (Jandl *et al.*, 1956) in Case II. (Hodgkin's disease with hypersplenism and immunoglobulin deficiencies.)

Results of surface counting, showing increased splenic red cell destruction.

●——● Spleen. ○——○ Liver.

FIG. 2.—Photomicrograph ( × 500) of spleen, removed at splenectomy in Case II. This shows reticulum cell hyperplasia. No mirror-image giant cells in this section.

Fig. 1.

Fig. 2.

Hoffbrand.

investigation, to have a frank reduction of the serum gamma globulin level. He was, also, the only one of these cases to have a clinically apparent haemolytic anaemia. This stresses the probable significance of the association of immunoglobulin deficiency and haemolytic anaemia.

Most reported cases of idiopathic acquired hypogammaglobulinaemia, with haematological abnormalities, have had splenomegaly and reticulum cell hyperplasia (Prasad *et al.*, 1957). There is independent evidence of a close relationship between the haematological and histological changes. Von Haam and Awny (1948), examining 102 spleens removed for essential hypersplenism, found reticulum cell hyperplasia, generally proportional in degree, to the severity of the disease.

The reticulum cell hyperplasia, found in many cases of idiopathic acquired hypogammaglobulinaemia (Martin, 1962) and in Case II, reported here, may represent a block in stem cell maturation. On antigenic stimulation in such cases, there is proliferation of reticulum cells in the spleen and lymph nodes, but no plasma cell or secondary germinal follicle formation (Gitlin, 1963).

The rarity of immunoglobulin deficiencies in Hodgkin's disease (Videbaek, 1962, personal communication), makes it unlikely that haemolytic anaemias in this condition are often associated with such a dysproteinaemia. However, immunoglogulin studies should be undertaken whenever unexplained haemolytic anaemia, hypersplenism, or reticulum cell hyperplasia is found in Hodgkin's disease.

SUMMARY

Two cases of Hodgkin's disease with frank haemolytic anaemia are reported. One case had reticulum cell hyperplasia and hypersplenism, which responded to splenectomy. Both cases had deficient immunoglobulin levels, a probably significant association as haemolytic anaemia and hypersplenism are not uncommon in idiopathic acquired hypogammaglobulinaemia. Immunoglobulin levels should be estimated whenever otherwise unexplained haemolytic anaemia, hypersplenism, or reticulum cell hyperplasia, is found in Hodgkin's disease.

I wish to thank Dr. T. A. J. Prankerd and Dr. F. V. Flynn for much helpful advice and criticism. I am also indebted to Dr. J. F. Soothill for the immunoglobulin estimations, to Dr. Peter Sutton for details of the pathological findings, to Dr. T. St. M. Norris for information about Case II, and to Dr. A. J. Bowdler and Dr. Peter Toghill for the radiochromium studies.

This report is based on work done during the tenure of a grant from the British Empire Cancer Campaign.

REFERENCES

Bowdler, A. J. and Prankerd, T. A. J.—(1962) *Brit. med. J.*, i, 1169.
Gitlin, D.—(1963) *New Engl. J. Med.*, **268**, 208.
Hoffbrand, B. I.—(1964) *Brit. med. J.*, in press.
Jandl, J. H., Greenberg, M. S., Yonemoto, R. H. and Castle, W. B.—(1956) *J. clin. Invest.*, **35**, 842.
Martin, N. H.—(1962) *Proc. R. Soc. Med.*, **55**, 398.
Prasad, A. S., Reiner, E. and Watson, C. J.—(1957) *Blood*, **12**, 926.
Soothill, J. F.—(1962) *J. Lab. clin. Med.*, **59**, 859.
Thompson, E. N. and Johnson, R. S.—(1962) *Post Grad. med. J.*, **38**, 292
Von Haam, E. and Awny, A. J.—(1948) *Amer. J. clin. Path.*, **18**, 313.